# Gastric lesions associated with the infection of Anisakidae nematodes in a dwarf sperm whale *Kogia sima* (Owen, 1866) stranded in the north coast of Brazil

**DOI:** 10.1016/j.ijppaw.2024.101028

**Published:** 2024-12-04

**Authors:** Gisele C.C. Seade, David F. Conga, Tiago S. Santos, Marcio O. Moura, Diana M. de Farias, Lana O. Silva, Alexandra F. Costa, Tayanna M. Amaral, Maura M. de Souza, Renata Emin-Lima, Alessandra Scofield, Gabriela Riet-Correa, Valíria D. Cerqueira, Pedro S. Bezerra Júnior

**Affiliations:** a Universidade da Amazônia (UNAMA), Rua Rosa Vermelha, 335 - Aeroporto Velho, CEP: 68010-200, Santarém, Pará, Brazil; b Grupo de Medicina da Conservação e Saúde Única, Instituto de Desenvolvimento Sustentável Mamirauá (IDSM), Estrada Do Bexiga, 2584, CEP: 69553-225, Tefé, Amazonas, Brazil; c Universidade Federal Do Pará (UFPA), BR 316, Km 61 - Saudade II - Cristo Redentor, CEP: 68740-970, Castanhal, Pará, Brazil; d Universidade da Amazônia (UNAMA), Avenida Alcindo Cacela, 287 – Umarizal, CEP: 66060-902, Belém, Pará, Brazil; e Instituto Bicho D'agua: Conservação Socioambiental (IBD), Cj. Cohab, Gleba 2, Tv. B, 183, Marambaia, CEP: 66623-311, Belém, Pará, Brazil

**Keywords:** *Anisakis*, *Pseudoterranova*, *Skrjabinisakis*, Gastric ulcers, *Kogia*

## Abstract

The present study aimed to describe gastric lesions associated with parasitism by different nematodes of the family Anisakidae in a stranded specimen of dwarf sperm whale (*Kogia sima*) on the northeast coast of the State of Pará, northern Brazil. Specimens of helminths and samples of stomach tissue were collected from a dwarf sperm whale, stranded on Humaitá beach, State of Pará, Brazil. Stomach showed areas of erosion and ulcers, with the mucosa covered by fibrinonecrotic material containing bacteria and inflammatory infiltrate predominantly comprising polymorphonuclear cells. Granulomas were also found in the submucosa, characterized by central areas of necrosis and hemorrhage, and cross sections of nematodes were observed. Fourth-stage larvae of the genus *Pseudoterranova*, two morphotypes of fourth-stage larvae of the genus *Anisakis* and adult specimens of *Skrjabinisakis paggiae* were morphologically identified. Molecular and phylogenetic analyzes confirmed the identity of the partial sequences of the cox2 mtDNA gene for adult specimens of *S. paggiae*. This study contributes to our understanding of the distribution of different of anisakids in *K. sima* and about the gastric lesions associated with these nematodes, in addition to expanding the knowledge about the occurrence of this aquatic mammal recorded for the first time in the northern region of Brazil.

## Highlights

• *Kogia sima* was parasitized by three larvae species and one adult of anisakids infecting the gastric mucosa.
• Gastric ulcerative and erosions lesions associated with anisakids infection were observed.
• Cox-2 mtDNA sequences from specimens analyzed revealed 99.67% identity with partial Cox-2 mtDNA sequences from *A. paggiae*.
• *Kogiasima* was found in a peculiar geographic location, the estuary region of the Pará river.

## Graphical abstract

**Figure:**
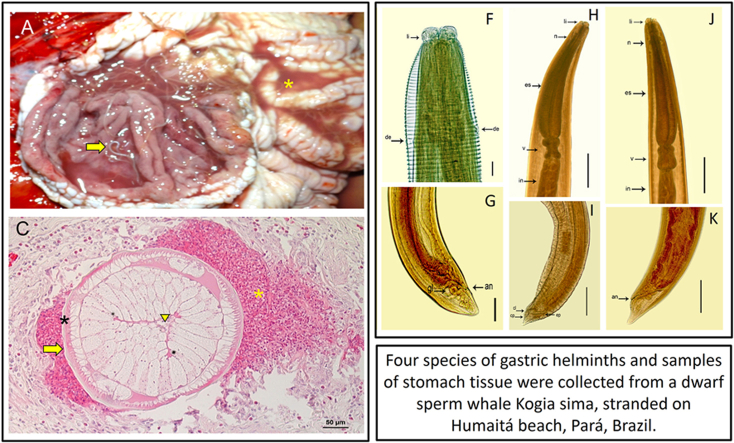

## 1 Introduction

Parasitic helminths are the most numerous metazoans in aquatic ecosystems, forming ecological interrelationships within the food chain between fish, copepods, and mammals (Palomba et al., 2023). Among these helminths, the nematodes of the family Anisakidae parasitize the viscera and muscles of several species of fish and other aquatic-associated animals which act as intermediate/paratenic hosts (Kuzmina et al., 2014; Shamsi et al., 2017; Pons-Bordas et al., 2020).

According to Ángeles-Hernández et al. (2020) the family Anisakidae comprises seven genera including *Anisakis*, *Contracaecum*, *Mawsonascaris*, *Pseudoterranova*, *Phocascaris*, *Sulcascaris* and *Terranova*. However, nowadays morphologic analyses and molecular genetics data studies have proposed to restore taxonomics status of the subgenus *Skrjabinisakis* (Mozgovoy, 1953) for the genus level and to assign the species *Anisakis brevispiculata* (Dollfus, 1966), *A. paggiae* Mattiucci et al. (2005) and *A. physeteris* (Baylis, 1923) to genus *Skrjabinisakis* (Safonova et al., 2021). In addition, Bao et al. (2023) proposed the resurrection of the genus *Phocanema*, with *Ph. decipiens* (sensu stricto) as the type species, to encompass *Ph. decipiens*, *Ph. azarasi*, *Ph. bulbosa*, *Ph. cattani* and *Ph. krabbei*.

*Kogia sima* is a small cetacean of the Family Kogiidae, distributed in all oceans and tropical seas with a warm climate and relatively frequent along the coast of Brazil (Maia et al., 2001) and anisakid infections have been diagnosed in this marine cetacean in Brazilian waters in the past (Di Azevedo et al., 2015, 2017). The present study aimed to describe gastric lesions associated with parasitism by different nematodes of the family Anisakidae in a stranded specimen of dwarf sperm whale (*Kogia sima*) on the northeast coast of the State of Pará, northern Brazil.

## 2 Material and methods

In the late afternoon of October 4, 2018, an adult female dwarf sperm whale (*Kogia sima*) was found on Humaitá beach, located in Marajó bay, in the municipality of Colares, State of Pará, northern region of Brazil. After some attempts to extricate the animal, it died during the night. Five hours after the death was recorded, the corpse was transported by the Department of the Environment of the municipality of Colares, under the supervision of the Brazilian Institute of the Environment and Renewable Natural Resources (IBAMA), to the Animal Pathology Laboratory (LAPATO) of the Institute of Veterinary Medicine of Universidade Federal do Pará, campus Castanhal, where the necropsy was carried out on the morning of October 5th by the laboratory team and collaborators from the Instituto Bicho D'agua (IBD). The conservation level of the carcass was evaluated according to criteria established by Pugliares et al. (2007). After external examination and necropsy, fragments of various organs were collected and fixed in 10% buffered formalin for histopathological analysis.

Additionally, live helminths found mostly in the main stomach but also in the forestomach were collected, washed in saline solution and separed in two groups of samples with larvae and adult helminth specimens. One group of samples was criopreserved for DNA extraction while of the other group was fixed using formalin-aceto-alcohol (FAA) solution, subsequently clarified in 50% Amman lactophenol and classified taxonomically according to the morphological keys of Mattiucci et al. (2005) and Safonova et al. (2021).

Genomic DNA was extracted from helminth specimens using ReliaPrep™ gDNA Tissue Miniprep System kit (PROMEGA, Madison, USA) according to manufacturer's instructions with modifications in the incubation time in proteinase K, which was performed overnight. Amplification of anisakid DNA was performed by Polymerase Chain Reaction (PCR) following the protocol of Mattiucci et al. (2011), with modifications. The primers 211F (5′-TTT TCT AGT TAT ATA GAT TGR TTT AT-3′) and 210R (5′-CAC CAA CTC TTA AAA TTA TC-3′) were used to amplify the 654 bp fragment of the mitochondrial cytochrome *c* oxidase subunit II (cox2 mtDNA) gene spanning the mtDNA nucleotide position 10,639–11,248, as defined in *Ascaris suum* [GenBank X54253] (Nadler and Hudspeth, 1998).

The amplification solution contained buffer (100 mM Tris-HCl, pH 8.5, 500 mM KCl), 50 mM MgCl_2_, 5 units of Taq DNA polymerase (Ludwig Biotec®, Alvorada, Brazil), 1 mM of each deoxynucleotide (dATP, dGTP, dCTP, and dTTP), 30 pmoles of each primer, and 5 μL sample DNA in a total reaction volume of 25 μL.

The reactions were performed in a gradient thermocycler (Veriti 96 Well Thermal Cycler, Applied Biosystems, Foster City, USA) using the following conditions: 94 °C for 3 min, 35 cycles of 94 °C for 30 s, 46 °C for 60 s, and 72 °C for 90 s, with a final extension of 72 °C for 10 min.

PCR products were analyzed by horizontal electrophoresis in 0.8% agarose gel containing non-mutagenic GelRed® (Biotium, Hayward, CA, USA) stain. The length of the amplified products was estimated using a pattern of 100 base pairs (100 Base-Pair-Ladder, Ludwig Biotec™), and these products were visualized in a transilluminator coupled with a photo documentation system (Image LabTM V. 5.2, Bio-Rad). The amplified products were purified with a commercial kit (EZ-10 Spin Column PCR Products Purification Kit) and sequenced in an automatic sequencer (ABI Prism 3500 Genetic Analyzer, Applied Biosystems™), according to the ABI PRISM Big Dye Terminator sequencing protocol.

The consensus sequences were submitted to BLASTn (https://www. ncbi.nlm.nih.gov) and compared with sequences of species of the genus *Skrajabinisakis* stored in GenBank. For sequence editing, the Geneious v.10.0.6 program (Kearse et al., 2012) was used, and for generating sequence clusters based on similarity, the CD-HIT program (Fu et al., 2012) was used, totaling the 42 most representative sequences. Subsequently, the database sequences and the sequences obtained in this study were aligned in the Mafft v.7 program (Larsson, 2014) implemented within the Aliview v.1.17.1 program (Katoh and Standley, 2013).

The genetic similarity was calculated using the Kimura two-parameter model and MEGA 7 software (Kumar et al., 2016). Multiple alignments were performed and a phylogenetic tree was constructed using the *Neighbor-Joining* method with 1000 bootstrap replicates. Statistical values with supports lower than 70% were ignored and *Pseudoterranova* spp. sequences were used as the outgroup.

## 3 Results

### 3.1 Necropsy and histopathological analysis

**Fig. 1:**
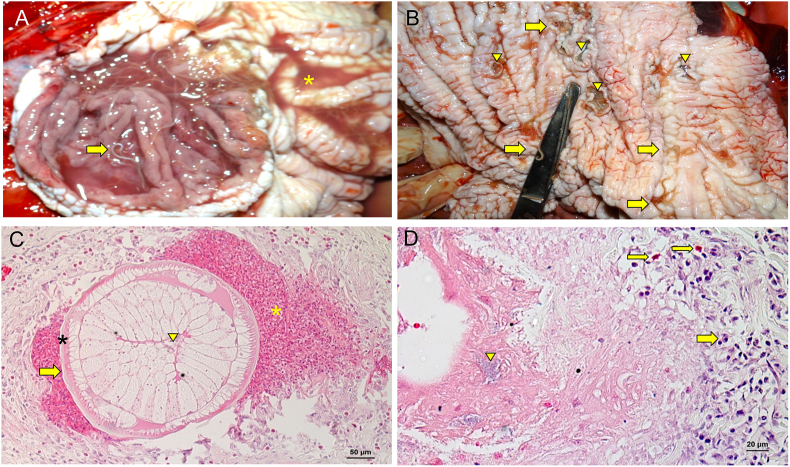
Parasitic gastritis associated with anisakids. (A) Gastric mucosa with a large number of anisakid nematodes (arrow) and bloody liquid content (asterisk). (B) Gastric mucosa with anisakid nematodes (arrow) and erosions and ulcers in nonglandular mucosa (arrowhead). (C) Gastric mucosa with cross sections of nematode bordered by necro-hemorrhagic material (yellow asterisk); note parasite cuticle (arrow), muscular layer (black asterisk), and intestinal lumen (arrowhead). (D) Gastric mucosa showing granulomatous inflammation in the submucosa with basophilic bacterial colonies (arrowhead) in the midst of necrosis bordered by moderate infiltrate of mononuclear cells (thick arrow) and eosinophils (thin arrow).

The carcass of the whale *Kogia sima* measured 2.5 m in length and was classified as code 2, based on its recent death history and in well conserved state of the cadaver and cavity organs (Pugliares et al., 2007). The external examination showed a slight swelling under the right eye and several excoriations on the skin. In total 208 nematode specimens were found in the stomach, measuring 10.53–26.69 mm in length, moving through the bloody liquid content (Fig. 1A). We also observed several erosions and ulcers in glandular and nonglandular mucosa of the stomach, with diameters ranging from 0.5 to 2.0 cm (Fig. 1B). There was a dark mucous content in the initial third portion of the small intestine, and a dark red to blackish pasty material in the final portion. The lungs were dark red and moist.

In the histopathological analysis, the stomach exhibited areas of erosion and ulcers with the mucosa covered by fibrinonecrotic material, sometimes containing basophilic bacteria (cocci and bacilli), and underlying inflammatory infiltrate predominantly comprising polymorphonuclear cells (Fig. 1D). We also found granulomas in the submucosa, characterized by central areas of necrosis and hemorrhage, exhibiting cross sections of nematodes (Fig. 1C). These areas were bordered by moderate-to-severe inflammatory infiltrate, comprising eosinophils, macrophages, multinucleated giant cells, lymphocytes, and fibrous connective tissue. The liver displayed diffuse and evident congestion as well as hepatocytes with diffuse vacuolization of the cytoplasm. The heart and kidneys showed diffuse and intense congestion, and lungs were edematous.

### 3.2 Morphological analysis of nematodes

**Fig. 2:**
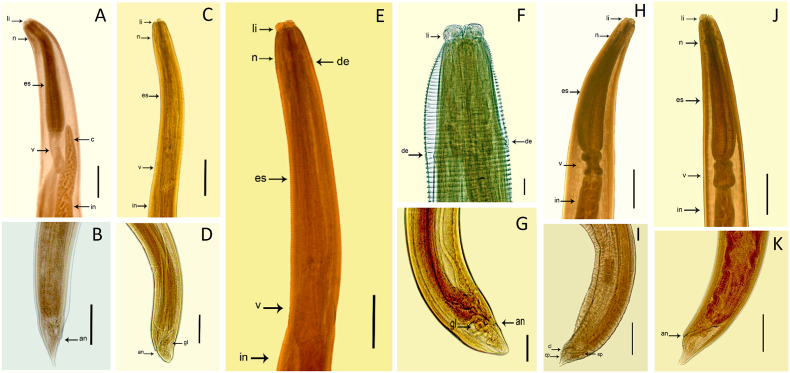
Morphology of the anterior and posterior ends of four anisakids. **(A; B)***Pseudoterranova* fourth-stage larva, scale bar = 0.5 mm **(C; D)** fourth-stage *Anisakis* sp. M1, scale bar = 0.2 mm **(E; F; G)** fourth-stage *Anisakis* sp. M2, scale bar = 0.1 mm **(H; I)***S. paggiae*, male adult. **(J; K)***S. paggiae*, adult female, scale bar = 0.5 mm: **(li)** lips, **(n)** nerve ring **(de)** deirids, **(es)** esophagus, **(v)** ventriculus, and **(in)** intestine, **(c)** intestinal cecum, **(an)** anal opening, **(gl)** rectal glands, **(cp)** caudal papillae, **(ep)** spicules, **(cl)** cloacal opening.

The first group of nematodes (analysis based on ten specimens, Table 1) were morphologically compatible with fourth-stage larvae of the genus *Pseudoterranova*, exhibiting the following features: three well-developed lips, two ventrolateral lips, one dorsal lip and interlabial teeth. Nerve ring located in the anterior third of the muscular esophagus. Ventriculus was located posteriorly to the esophagus, the intestinal cecum was observed surpassing slightly the ventriculus and the presence of two cloacal glands was visible near the anus. Vulvar opening was visible in the middle of the body and the specimens presented a pointed conical tail (Fig. 2A and B).

**Table 1:** Morphological and morphometric (μm) data of four group anisakids collected from a dwarf sperm whale in the state of Pará, Brazil.

Parameters	Group anisakids				
	*Pseudoterranova* sp. (n = 10)	*Anisakis* sp. M1 (n = 10)	*Anisakis* sp. M2 (n = 10)	*Skrjiabinisakis paggiae* (n = 20)	
	L4	L4	L4	Adult female	Adult male
Body length	14 (10–26)	19 (17–27)	20 (14–24)	19 (16–21)	19 (17–20)
Deirids to anterior end	absent	absent	0.3 (0.2–0.4)	absent	absent
Esophagus length	1.5 (1–2)	1.8 (0.9–2.0)	1.6 (1.0–2.0)	1.9 (1.7–2.1)	1.9 (1.7–2.0)
Esophagus width	0.2 (0.1–0.2)	0.2 (0.1–0.3)	0.1 (0.1–0.2)	0.4 (0.3–0.4)	0.4 (0.3–0.4)
Ventriculus length	0.5 (0.4–0.7)	0.6 (0.4–0.8)	0.6 (0.4–0.8)	0.4 (0.3–0.4)	0.4 (0.3–0.4)
Ventriculus width	0.15 (0.1–0.2)	0.2 (0.1–0.3)	0.1 (0.1–0.2)	0.2 (0.1–0.3)	0.2 (0.1–0.2)
Cecum length	0.6 (0.4–0.8)	absent	absent	absent	absent
Right spicule length	absent	absent	absent	–	0.2 (0.1–0.2)
Left spicule length	absent	absent	absent	–	0.2 (0.1–0.2)
Tail length	0.2 (0.2–0.4)	0.2 (0.1–0.3)	0.1 (0.1–0.2)	0.2 (0.1–0.2)	0.2 (0.1–0.2)
Ratio (esophagus + ventriculus)/body length	14.3%	12.6%	11.0%	12.1%	12.1%

∗The arithmetic mean value and minimum and maximum values in parentheses.

The morphology of the second group of nematodes (analysis based on ten specimens, Table 1) was compatible with fourth-stage larvae of the genus *Anisakis*, presenting the following characteristics: all of which had three lips, two ventrolateral, one dorsal lip and a nerve ring located in the anterior third of the esophagus at the anterior end. Absence of intestinal cecum. The ventriculus was short and located posteriorly to the esophagus, three cloacal glands and a blunt tail were observed (Fig. 2C and D). We identified these specimens as morphotype 1 (M1) fourth-stage larvae of the genus *Anisakis*.

The third group of nematodes (analysis based on ten specimens, Table 1) was morphologically compatible with fourth-stage larvae of the genus *Anisakis* and exhibited the following features: three lips, two ventrolateral, one dorsal lip, and nerve ring was in the anterior third of the esophagus and deirids were observed located laterally and at the level of the nerve ring, muscular esophagus; short ventriculus located posterior to the esophagus, absence of intestinal cecum and in the final posterior part, five cloacal glands and a blunt tail were observed (Fig. 2E, F, G). The presence of deirids in these specimens represented a morphological difference compared to those assigned to the *Anisakis* M1 morphotype, therefore these specimens were identified as morphotype 2 (M2) fourth-stage larvae of the genus *Anisakis*.

The fourth group of nematodes (morphometry based on twenty specimens, Table 1) included helminths that were morphologically compatible with adult specimens of *Skrjiabinisakis paggiae* (Mattiucci et al., 2005) because they exhibited the following features: visible reproductive organs, three lips at the anterior end (two ventrolateral and one dorsal), a claviform esophagus, a nerve ring located in the anterior third of the esophagus, a violin-shaped ventriculus, a short conical tail, the male specimens showed pedunculated caudal papillae, ten pairs of pre-cloacal papillae, six post-cloacal papillae, the presence of four rectal glands and a subterminal anus (Fig. 2H, I, J, K).

### 3.3 Molecular and phylogenetic analyses of adult specimens

Genomic DNA was extracted from three pools of larvae and two adult helminth specimens that were morphologically compatible with the morphotypes *Pseudoterranova* (n = 16), *Anisakis* M1 (n = 13), *Anisakis* M2 (n = 8) and with adult specimens of *S. paggiae*, respectively. In addition, it is important to emphasize that DNA extraction was carried out in cryopreserved adult helminth specimens and pools of larvae fixed in FAA solution, as there was morphological deformation of the cryopreserved larvae, making it impossible to identify the morphotypes.

**Fig. 3:**
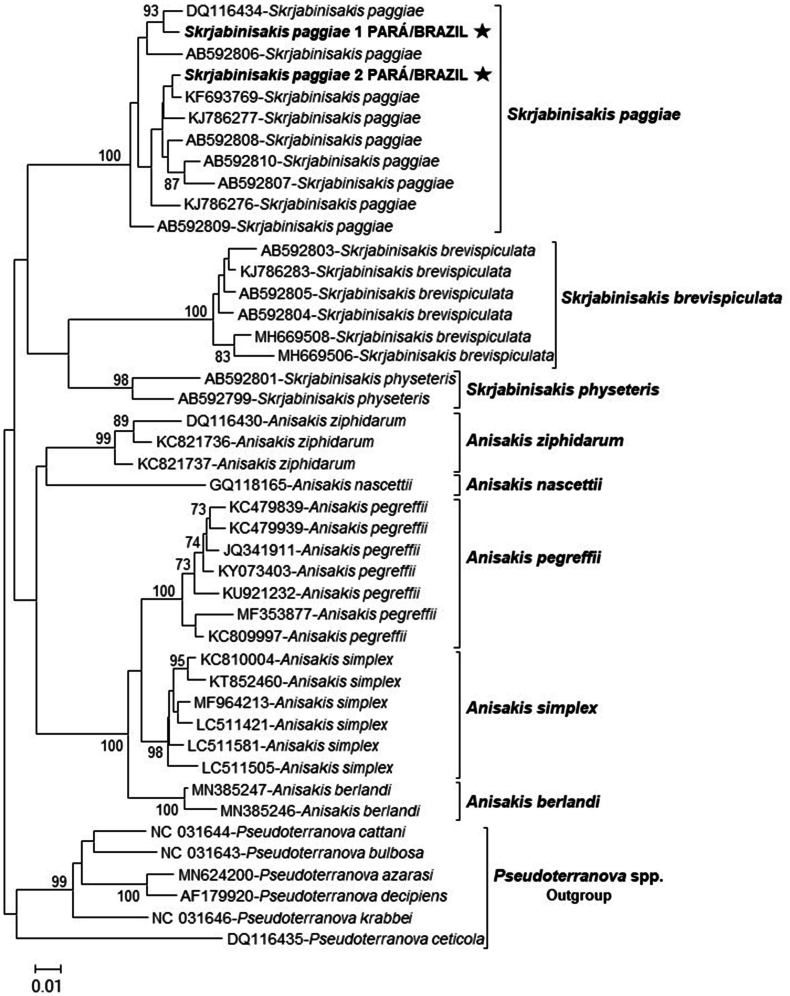
Phylogenetic tree built using the *Neighbor-Joining* method with two partial sequences of the cox2 mtDNA gene of *S. paggiae* isolates in *Kogia sima* (highlighted) stranded in the north coast of Brazil and 42 sequences of anisakids deposited in GenBank (accession numbers shown). The bootstrap percentage of trees in which the associated taxa clustered together is shown next to the branches. The scale bar indicates the number of substitutions per site. The sequences from *Pseudoterranova* spp. were used as an outgroup.

The amplification of the partial cox2 mtDNA gene sequences occurred only in the DNA samples of adult helminth specimens criopreserved and the two sequences obtained exhibited a 97%–99.67% identity with other partial cox2 mtDNA gene sequences of *S. paggiae* available in GenBank (AB592810.1, AB592808.1, EU560910.2, KC342895.1, KJ786280.1, AB592807.1, KJ786279.1, KJ786276.1, AB592806.1, KC821731.1, KC342896.1 and AB592809.1). Through phylogenetic analysis, it was possible to confirm the identity of the partial sequences of the cox2 mtDNA gene of *S. paggiae* (Fig. 3). The two sequences obtained were grouped into a clade together with nine other sequences of *S. paggiae* isolated from *Kogia breviceps* in the USA (DQ116434.1) and in the Philippines (KJ786277.1, KJ786276.1), from *S. paggiae* collected from *K. sima* in northeastern Brazil (KF693769.1), and from *S. paggiae* collected from *Beryx splendens* in Japan (AB592806.1, AB592807.1, AB592808.1, AB592809.1, AB592810.1).

## 4 Discussion

Parasites are intrinsic components of marine ecosystems and play complex roles in host-parasite interactions (Timi and Poulin, 2020). The use of biological samples from stranded marine mammals contributes to monitoring biodiversity, detecting environmental changes, assessing the health of marine organisms and consequently human health. By combining molecular analyzes and morphological data on nematodes, we highlighted an important diversity of nematodes of the family Anisakidae compromising the stomach of the dwarf sperm whale *K. sima*.

The cetacean analyzed in the present study was found in the estuary of the Pará river, a peculiar geographic location where whale strandings are not frequent (Muñoz-Hincapié et al., 1998; Siciliano et al., 2008; Moura et al., 2016; Di Azevedo et al., 2017). The Pará river is part of a large tropical estuarine system dominated by tides. Thus, it is responsible for the transport of large amounts of water from the ocean to this estuary, which might have facilitated the drift of the already weakened whale to the coastal region during the high tide period (Prestes et al., 2017; Carneiro et al., 2020).

Gastric ulcers in marine mammals are frequently associated with the presence of a high load of nematodes. Ulcers can range from acute, with congestion, edema, hemorrhage, and eosinophilic inflammatory infiltrate, to chronic, with fibrosis and granuloma formation (Motta et al., 2008; Shamsi et al., 2019). Although the samples were collected some time after the death of whale, due to the remoteness of the place, the analysis indicated an anisakid-associated chronic infection and acute lesions at different stages of development. Although we had a limitation in obtaining genetic sequences of all the Anisakidae specimens observed, we were able to identify morphologically and morphometrically three different morphotypes of fourth-stage larvae and one species of adults.

The fourth-stage larvae of *Pseudoterranova* sp. were characterized morphologically by a long ventriculus, and the presence of an intestinal cecum but no ventricular appendix. Adult species of genus *Pseudoterranova* have been reported to infect the stomach of cetaceans *Kogia breviceps* (Santos and Lodi, 1998; Cipriani et al., 2024), *Delphinapterus leucas*, *Monodon monoceros*, *Stenella longirostris* (Di Azevedo et al., 2017), *Phocoena, Delphinus delphis, Lagenorhynchus albirostris, Balaenoptera acutorostrata,* and *B. musculus* (Abollo and Pascual, 2002; Solís et al., 2006). In addition to this record of fourth-stage larvae of *Pseudoterranova* infecting *K. sima* in the South Atlantic, adult specimens of *Pseudoterranova ceticola* have been reported on this same host in southeastern Atlantic coasts of USA, Gulf of Mexico and Caribbean Sea (Cavallero et al., 2011).

Although the amplification of the partial cox2 mtDNA gene sequence was not possible from *Anisakis* sp. M1 larvae, the morphological characteristics of these specimens are compatible with *Skrjabinisakis brevispiculata*. This species is distributed in the South and Central Atlantic, observed in low prevalence and intensity, in fish, squid or other species of the family Cranchiidae, intermediate hosts that are part of the diet of the whales *K. sima* and *K. breviceps* of the Atlantic Ocean in Florida, Caribbean Sea, South Africa and the Philippines (Dos Reis and Luque, 2016; Mattiucci et al., 2018a; Shamsi et al., 2012). *S. brevispiculata* has also been recently observed in cetaceans *Phocoena* and *K. breviceps* (Cipriani et al., 2022a, 2024).

A characteristic of the specimens categorized as *Anisakis* M2 in the present study was the presence of deirids. This feature has been described in L4 larvae of *Skrjabinisakis physeteris* (Molina-Fernández et al., 2018) and *Anisakis pegreffii* (Mladineo et al., 2023). Previous records of adult specimens of *S. physeteris* in Brazil were observed in oceanic cetaceans *Balaenoptera acutorostrata*, *Globicephala melas*, *Hyperodon ampullatus*, *Kogia breviceps*, *Physeter macrocephalus* and *Ziphius cavirostris* (Santos and Lodi, 1998). There are also studies reporting larvae of *S. physeteris* in frigate tuna (*Auxis thazard*) on the Brazilian coast (Iñiguez et al., 2009).

Molecular and phylogenetic analyzes confirmed the identity of the partial sequences of the cox2 mtDNA gene for adult specimens of *S. paggiae*. These analyzes contribute to investigations about anisakid infections in aquatic mammals, as studies suggest that cox2 mtDNA is a suitable genetic marker for the analysis of the genetic structure of species of the family Anisakidae (Mattiucci et al., 2018b; Cipriani et al., 2022b). In addition, we report, for the first time, the identification of *S. paggiae* sequenced from *K. sima* in the north coast of Brazil. It is important to emphasize that these *S. paggiae* sequences were obtained from cryopreserved anisakids. There was no amplification of the partial cox2 mtDNA gene sequences in DNA samples extracted from anisakids larvae conserved in FAA solution and this may have occurred due to the presence of PCR inhibitors in DNA samples or degradation these samples. The nematodes *S. paggiae* are apparently limited to definitive hosts of the genus *Kogia* (Cipriani et al., 2024) and the specimens found in this study were smaller in total size than those described in *K. sima* by Di Azevedo et al. (2015, 2017).

In this study, the *K. sima* whale was parasitized by different anisakids in the larval stage and by *S. paggiae* adult specimens sympatrically infecting the gastric mucosa, which indicates a constant infection. Previous studies described infections by *A. typica*, *A. ziphidarum*, *S. brevispiculata*, and *S. paggiae* in *K. sima* host in Brazil (Di Azevedo et al., 2017). In the Philippines, *A. typica, S. brevispiculata* and two other species genetically close to *S. paggiae* and *A. ziphidarum* were identified in *K. sima* host (Quiazon et al., 2013). Thus, the data presented corroborate that this sympatry between anisakids in *K. sima* occurs, reported this time in the northern region of Brazil.

## 5 Conclusion

In conclusion, this study contributes to our understanding of the distribution of different of anisakids in *K. sima* and about the gastric lesions associated with these nematodes, in addition to expanding the knowledge about the occurrence of this aquatic mammal recorded for the first time in the northern region of Brazil.

## CRediT authorship contribution statement

**Gisele C.C. Seade:** Writing – original draft, Data curation, Conceptualization. **David F. Conga:** Writing – original draft, Validation, Methodology, Investigation, Formal analysis, Data curation, Conceptualization. **Tiago S. Santos:** Visualization, Validation, Resources, Methodology, Investigation, Formal analysis. **Marcio O. Moura:** Visualization, Validation, Resources, Methodology, Investigation, Formal analysis. **Diana M. de Farias:** Visualization, Validation, Resources, Methodology, Investigation, Formal analysis. **Lana O. Silva:** Visualization, Validation, Resources, Methodology, Investigation, Formal analysis. **Alexandra F. Costa:** Visualization, Validation, Resources, Methodology, Investigation, Formal analysis. **Tayanna M. Amaral:** Validation, Supervision, Methodology, Investigation, Formal analysis. **Maura M. de Souza:** Visualization, Validation, Methodology, Investigation, Formal analysis. **Renata Emin-Lima:** Visualization, Validation, Methodology, Investigation, Formal analysis, Gisele Cristine Castro Seade, Visualization, Supervision, Formal analysis, Conceptualization. **Alessandra Scofield:** Visualization, Validation, Resources, Methodology, Investigation, Formal analysis, Data curation, Conceptualization. **Gabriela Riet-Correa:** Visualization, Validation, Resources, Methodology, Investigation, Funding acquisition, Formal analysis, Data curation, Conceptualization. **Valíria D. Cerqueira:** Visualization, Validation, Supervision, Resources, Project administration, Methodology, Investigation, Funding acquisition, Formal analysis, Data curation, Conceptualization. **Pedro S. Bezerra Júnior:** Visualization, Validation, Supervision, Resources, Methodology, Investigation, Funding acquisition, Formal analysis, Data curation, Conceptualization.

## Ethical statement

This study was authorized under number 38130–1 ICMBio-SISBIO. Our investigation in this study has not included animal experiment.

## Funding

This study has been supported by 10.13039/501100002322Coordination for the Improvement of Higher Education Personnel - Brazil (10.13039/501100002322CAPES), Gisele C. Seade is funded by the 10.13039/501100002322CAPES doctoral-grant.

## Declaration of competing interest

There are no relationships or support that might be perceived as constituting a conflict of interest.
