# Supplementary material for: Gastric lesions associated with the infection of Anisakidae nematodes in a dwarf sperm whale Kogia sima (Owen, 1866) stranded in the north coast of Brazil
Source: Int J Parasitol Parasites Wildl. 2024 Dec 4;26:101028. doi: 10.1016/j.ijppaw.2024.101028 (PMC12130977; doi:10.1016/j.ijppaw.2024.101028)
Supplement: Multimedia component 1 [file mmc1.docx]

**Table 1**- Morphological and morphometric parameters of four anisakids collected from a dwarf sperm whale in the state of Pará/Brazil. Mean measurements in millimetres and range between parentheses

|  | *Pseudoterranova*  (n=10) | *Anisakis* M1 (n=10) | *Anisakis* M2 (n=10) | *Skrjiabinisakis paggiae*  (n=20) | |
| --- | --- | --- | --- | --- | --- |
| Parameters | L4 | L4 | L4 | Adult female | Adult male |
| Body length | 14 (10–26) | 19 (17–27) | 20 (14–24) | 19 (16–21) | 19 (17–20) |
| Deirids to anterior end | absent | absent | 0.3 (0.2–0.4) | absent | absent |
| Esophagus length | 1.5 (1–2) | 1.8 (0.9–2.0) | 1.6 (1.0–2.0) | 1.9 (1.7–2.1) | 1.9 (1.7–2.0) |
| Esophagus width | 0.2 (0.1–0.2) | 0.2 (0.1–0.3) | 0.1 (0.1–0.2) | 0.4 (0.3–0.4) | 0.4 (0.3–0.4) |
| Ventriculus length | 0.5 (0.4–0.7) | 0.6 (0.4–0.8) | 0.6 (0.4–0.8) | 0.4 (0.3–0.4) | 0.4 (0.3–0.4) |
| Ventriculus width | 0.15 (0.1–0.2) | 0.2 (0.1-0.3) | 0.1 (0.1–0.2) | 0.2 (0.1–0.3) | 0.2 (0.1–0.2) |
| Cecum length | 0.6 (0.4–0.8) | absent | absent | absent | absent |
| Right spicule length | absent | absent | absent | – | 0.2 (0.1–0.2) |
| Left spicule length | absent | absent | absent | – | 0.2 (0.1–0.2) |
| Tail length | 0.2 (0.2–0.4) | 0.2 (0.1–0.3) | 0.1 (0.1–0.2) | 0.2 (0.1–0.2) | 0.2 (0.1–0.2) |
